# Infant Feeding Apps and Digital Parenting in Australia: Qualitative Study

**DOI:** 10.2196/95934

**Published:** 2026-09-08

**Authors:** Diana Arabiat, Harrison See, Lisa Whitehead

**Affiliations:** 1 Maternal and Child Nursing Department, School of Nursing, The University of Jordan, Queen Rania Street, Amman, Amman, 11942, Jordan, 962 775471037; 2 Epidemiology, Biostatistics and Health Informatics, Public Health Institute, The University of Jordan, Amman, Amman, Jordan; 3 School of Nursing and Midwifery, Edith Cowan University, Joondalup, Western Australia, Australia; 4 ARC Centre of Excellence for the Digital Child, Brisbane, Queensland, Australia; 5 School of Arts and Humanities, Edith Cowan University, Joondalup, Western Australia, Australia

**Keywords:** data privacy, terms and conditions, digital apps, qualitative research, perspectives, risk perception, breastfeeding, first-time parents, Australia

## Abstract

**Background:**

Parents are increasingly using infant feeding apps (IFAs) for information and decision-making related to breastfeeding and other aspects of their baby’s care. Few studies have explored how users conceptualize data privacy risks in relation to IFAs and how concerns are balanced with app use.

**Objective:**

This qualitative study forms part of the Infant Feeding App project exploring how IFAs influence parent-child communication in Australian families. A focus of this investigation was to explore mothers’ understanding of the terms and conditions associated with the use of IFAs and their data privacy concerns.

**Methods:**

We included first-time parents of children aged 0 to 4 years who were breastfeeding at the time of the first interview and previously or currently used 1 or more IFAs. Twenty-four participants completed the first-round interview; of those, 22 returned for a second-round interview approximately 6 months later, and 7 participated in the focus group discussion. Reflexive thematic analysis was used to analyze the data.

**Results:**

This paper draws primarily on findings from the key category of “Data and/or Privacy Practices.” This category was further refined into 2 main themes: lack of awareness or acceptance of the terms and conditions associated with the mobile app and the risk mitigation behaviors mothers took to protect their privacy.

**Conclusions:**

Although mothers were aware of the privacy risks associated with IFAs, they nonetheless continued to engage with them, reflecting the broader growth in digital technology use. This paper has highlighted that privacy, a fundamental human right, is at risk when mothers share personal and child-related data through digital apps. Importantly, mothers’ willingness to use these applications despite perceived concerns highlights the tension between perceived utility and potential risks. A call for a move toward privacy by design, transparency in privacy policies, and an approach of nonalienation in the use of data is called for.

## Introduction

The number of infant feeding apps (IFAs) available to parents is growing, offering information to guide breastfeeding decisions and other aspects of care [1]. These apps typically enable the recording of feeding schedules and intake, sleeping patterns, growth metrics, and developmental milestones. The apps aim to offer timely support for women by enabling remote monitoring and information sharing throughout their pregnancy and the postpartum period [2-4]. IFAs have the potential to support timely access to information and have been reported as easy to use and create access to accurate information [5].

A recent randomized controlled trial in Turkey [5] showed that the use of IFAs influenced mothers’ breastfeeding experiences in a positive manner, with users reporting fewer breastfeeding-related difficulties and higher rates of exclusive breastfeeding. IFAs have been used as supportive tools for promoting early initiation and continuation of breastfeeding [6] and exchange of knowledge among parents [7,8]. Other studies suggest that the use of IFAs is perceived by some parents as an indicator of “good” parenting practices, or what has been termed “tracking-as-care” [9,10].

In Australia, parents have used IFAs to track their child’s activities and to assist them with decision-making [1,11]. IFAs have been described as enhancing parental knowledge acquisition [6] and improving parental confidence [1], yet consensus across the literature on the perceived value of IFAs remains difficult to achieve.

Concerns have been noted in relation to the quality and accuracy of the content and the functionality of IFAs [12,13]. These issues are compounded by the scarcity of empirical evidence on data confidentiality and privacy when using mobile and web apps [14]. The use of IFAs often entails continuous input of personal data, including feeding times, infant weight, sleep cycles, and health behaviors that may raise concerns about the opacity of data privacy policies and the extent to which parents understand or consent to data sharing practices [4]. To deliver their functions, IFAs collect a wide range of data that may include parent-related data and personally identifiable information, such as names, birth dates, email addresses, and location. Mortensen and Lomborg [15] have characterized this issue as the continuous generation of digital traces or “data selfies” through engagement with mobile self-tracking apps (p. 6013).

A further issue is the potential for third parties without the explicit knowledge of the user to access and use data. Lupton and Michael [16] found that people recognized the value that could be derived from their own personal data, as well as from the collective insights generated when their data were integrated into aggregated datasets, benefiting both themselves and others. However, they found that people expressed concerns or suspicion about how their data might be used by others and what ultimately happens to their data. Areas for further investigation in relation to these areas include the balance between sharing personal data, the value to the individual as a result, and data privacy.

Normalizing the datafication of everyday activities, such as sleeping data or feeding times (parent and child data), has been raised as a form of dataveillance [8,11]. The acceptance of apps such as IFAs can be argued to normalize the heightened surveillance of both women’s and children’s bodies [9]. The creation of a data trace across the lifespan is relatively new, with children becoming digital citizens sometimes before they are even born through pregnancy apps and social media postings [17]. Areas for further exploration include: when does personal dataveillance become too intrusive, and how do we balance the growing digitization of children in relation to the sharing, selling, and commodification of data?

While the use of IFAs may assist in developing innovative interventions to support infant feeding practices, the need to develop a more nuanced understanding of how parents perceive and manage issues of data privacy is needed. Therefore, this paper aimed to explore mothers’ understanding of the terms and conditions associated with the use of IFAs and their data privacy concerns.

## Methods

### Design

This study is part of an ongoing research project, “The parent-child communication implications of IFAs,” that aims to better understand the impacts of IFAs on parent-child communication among Australian families. The project broadly problematizes the inclusion of a screen as a novel component within early processes of infant feeding, but also acknowledges the potential benefits IFAs offer, especially to first-time parents. A key aim was to explore the motivations and concerns that support parents’ use of IFAs and the reasons why some parents might choose not to use them or might discontinue their use. Of particular relevance to this paper were motivations and concerns around privacy practices and terms and conditions.

The data used in this study were collected using a descriptive qualitative design comprising 2 rounds of interviews followed by focus group sessions. The data reported here are derived from 1 of the 2 focus groups conducted as part of a larger study. For inclusivity and flexibility in recruitment, participants were able to join either session according to their convenience. Data collection was concluded once thematic saturation was reached and when additional sessions were unlikely to yield new insights or generate further data.

Having multiple points of data collection created a degree of semilongitudinal data to capture parents’ evolving views, while focus groups have the potential to expand data as participants’ views may be supported or challenged by engaging in collective dialogue with others [18]. A semistructured interview guide was developed with questions designed to explore parents’ data privacy perspectives in the use of IFAs and in their broader digital parenting (Textbox 1).

Textbox 1. **Main questions from the interview guide.**

The following were the semistructured interview questions:

1. What factors influence new parents’ choice to use or not use infant feeding apps?
2. How do parents use infant feeding apps and what do they see as the benefits and drawbacks?
3. What understandings do parents have regarding the terms and conditions of their app use, and the resulting reuse of data and harnessing of data flows?
4. When, how, and why do parents start or end the use of infant feeding apps as part of their infant feeding practices?

### Participants and Recruitment

A total of 24 English-speaking participants were interviewed in 2024. Although recruitment targeted parents of infants aged 0 to 4 years, the actual age distribution was 1 to 10 months at the time of the first round of interviews. The IFA project collected qualitative data from participants through 2 rounds of semistructured interviews, followed by a series of focus groups. Of the initial 24 participants who took part in a first-round interview, 22 returned for a second-round interview approximately 6 months later, with 7 returning for a focus group. All participants were breastfeeding mothers of children at the time of the first round of interviews. A total of 46 interviews and 2 focus groups were conducted online between mid-2023 and mid-2024. Recruitment took place online via social media, predominantly through Facebook mothers’ groups, using the snowball method. Participants received an AUD $50 (US $33) gift card at each data collection point.

### Data Collection

The semistructured interviews were conducted online. The interviews were recorded with a digital audio-recording device and transcribed by the same researcher who performed the interviews. The interviews lasted a mean of 45 (SD 15) minutes.

### Analysis

Interviews and focus groups were transcribed and deidentified before coding in NVivo software (version 1.7.1; Lumivero). Before coding, the research team read through the interviews several times to become familiar with the transcripts and achieve deep immersion in the data. Subsequently, initial codes were systematically developed inductively and organized into subthemes and overarching thematic categories. Multiple researchers assisted in reviewing and examining the coded material within each theme and assisted in defining and refining the themes through the writing up of this study. Of the 5 final codes, the most relevant to this paper is number 4, “Data and/or Privacy Practices,” which was divided into further subcategories and themes.

To ensure rigor, interpretations of the coded data emerged from ongoing iterative discussions between all authors of the paper using reflexive thematic analysis [19]; as such, the authors acknowledge that their differing disciplinary and lifeworld perspectives influence their readings of the data, and that more comprehensive readings are potentially made accessible through interdisciplinary dialogue. This study employed reflexive thematic analysis to systematically elucidate patterns of meaning within parents’ narratives. The analytic process was strengthened by deliberate reflexivity, enabling critical examination of researchers’ preconceptions and fostering methodological transparency. Furthermore, the integration of surveillance theory as an interpretive framework informed the development of latent codes, thereby enhancing the conceptual depth and analytic rigor of thematic constructs.

In the context of this research, IFAs constituted any smartphone app that assisted parents to support or track their children’s feeding routine; overarchingly, this meant breastfeeding mothers; however, as the data are semilongitudinal, some participants were in the process of shifting to solid food. The most common IFAs used by parents were “Huckleberry” and “Solid Starts.” Of the 24 participants, 6 either did not regularly use IFAs at the time of the research or had never regularly used IFAs.

### Ethical Considerations

Data collection was undertaken by researchers from the Edith Cowan University node of the Australian Research Council Center of Excellence for the Digital Child under the oversight of Chief Investigator Professor Lelia Green. The project received approval from Edith Cowan University’s Human Research Ethics Committee (approval number 2022‐03677-GREEN) and was funded by the Australian Research Council Center of Excellence for the Digital Child (project number CE200100022).

All eligible participants were provided written information about the study, and their consent was obtained before data collection.

## Results

### Participant Characteristics

A total of 24 English-speaking participants were interviewed in 2024. Participant characteristics are reported in Table 1. Infants ranged in age from 1 month to 10 months at the time of the first interview, with balanced representation across genders and the highest proportion observed at 7 months. The duration of IFA use varied between 6 and 24 weeks at the time of the last interview and/or focus group. The majority of participants were based in Western Australia (n=13), with additional representation from New South Wales (n=4), Queensland (n=4), Victoria (n=2), and the Australian Capital Territory (n=1). Across the sample, patterns of parental engagement with IFAs were diverse. While some participants reported minimal or selective use, others actively engaged with multiple apps. Huckleberry, Solid Starts, and Wonder Weeks were the most frequently used.

**Table 1.** Overview of participant characteristics.

Family number	Mother’s pseudonym	Child age^a^ (gender)	Interview 1	Interview 2	Focus group
F01	Ariel	6-month-old female twins, has not used IFAs^b^ with current infants.	✓	✓	—^c^
F02	Helena	6-month-old female, has not used IFAs with current infant.	✓	✓	—
F03	Cassandra	6-month-old female, uses 1 IFA.	✓	✓	—
F04	Elspeth	1-month-old male, never used IFAs.	✓	✓	✓
F05	Sinead	10-month-old female, previously used one IFA, ceased at 3 months.	✓	✓	—
F06	Felicia	7-month-old female twins, has never used IFAs.	✓	✓	✓
F07	Hyacinth	5-month-old female, uses 2 IFAs, ceased another.	✓	✓	✓
F08	Cynthia	6-month-old male, uses 2 IFAs.	✓	✓	✓
F09	Lauretta	6-month-old male, uses 2 IFAs.	✓	✓	—
F10	Marion	7-month-old female, 1 IFA ceased at 5 months.	✓	✓	—
F11	Yennifer	10-month-old male, has never used IFAs.	✓	✓	—
F12	Lillian	6-month-old female, has never used IFAs.	✓	✓	—
F13	Lucia	5-month-old male, uses 2 IFAs.	✓	✓	✓
F14	Semra	8-month-old female, uses 2 IFAs.	✓	✓	—
F15	Morgana	7-month-old male, uses 1 IFA.	✓	—	—
F16	Lydia	7-month-old female, uses 1 IFA, ceased another at 3 weeks.	✓	✓	—
F17	Whitney	8-month-old female, uses 2 IFAs.	✓	✓	—
F18	Patty	8-month-old female, uses 1 IFA, ceased another at 4 months.	✓	—	—
F19	Bella	6-month-old male has never used IFAs.	✓	✓	✓
F20	Babette	5-month-old female, uses 1 IFA, ceased another at 4 months.	✓	✓	—
F21	Paris	6-month-old male, one IFA ceased at 5 months.	✓	✓	—
F22	Lisette	6-month-old female, uses 1 IFA, ceased another at 5 months.	✓	✓	—
F23	Kelsie	7-month-old male, uses 1 IFA, trialed one other.	✓	✓	—
F24	Bernadette	5-month-old female, uses 2 IFAs.	✓	✓	✓
Total	24 (mothers)	—	24	22	7

a Age of child at time of participating in first interview.

b IFAs: infant feeding apps.

c Not applicable.

### Overview

Across the entire project, 4 initial codes were generated through NVivo software (version 1.7.1). The final initial themes included the following:

1. Choice to adopt IFAs (1.A) or to avoid them (1.B).
2. Use of IFAs for record-keeping (2.A) or as a source of support (2.B).
3. Use of alternatives and other non-IFAs (3.A) or non-app modes of support (3.B).
4. Data privacy, including awareness or acceptance of terms and conditions (4.A), experiences or knowledge of risk-changing events, such as hacking or political issues (4.B), and ambivalence toward privacy (4.C).
5. Breastfeeding beyond the IFA context, which included positive (5.A) and negative aspects (5.B).

This paper specifically focuses on Theme 4: Data Privacy, which was refined through reflexive thematic analysis across 3 iterative cycles. During this process, the 3 initial codes were refined and reorganized into two main themes:

1. Awareness and/or acceptance of terms and conditions (subthemes included “data privacy laxity and normalization” and “benefits outweigh risk”).
2. Risk mitigation behaviors.

### Theme 1: Awareness and/or Acceptance of Terms and Conditions

#### Major Study Themes

A major theme that emerged from the mothers’ interviews and the focus group was mothers’ awareness of the privacy risks associated with IFAs, coupled with a pragmatic acceptance of the terms and conditions governing their use. Two subthemes reflected this. The first included “Data privacy laxity and normalization,” where mothers normalized and resignified the routine acceptance of surveillance and minimal engagement with terms and conditions. The second included “Benefits outweigh risks,” where the practical utility of these apps outweighed potential risks despite underlying discomfort.

#### Subtheme 1: Data Privacy Laxity and Normalization

Many participants expressed awareness of data collection and privacy risks associated with IFAs. However, they often described a sense of resignation or acceptance toward these practices, particularly in relation to lengthy and complex terms and conditions. Rather than carefully reviewing such documents, most participants reported clicking “accept” as part of everyday app use, recognizing the trade-off between convenience and privacy. Lydia, for example, shared:

*I just click accept, you know what I mean? I’m not going to put photos on there [...] my understanding was limited.*

[Lydia, 7-month-old daughter, uses one IFA, ceased another at 3 wk]

Other participants also commented:

*I don’t normally think about data collection and privacy and stuff in apps; it’s just so common, it’s supposed to be so much more helpful in our lives […] I don’t have time to sit there and read a 20-page long terms and conditions on your data collection processes*.

[Bernadette, 5-month-old daughter uses two IFAs]

*......I can’t say I’ve read the terms and conditions of every app that you use because who does? I go in with the assumption that nothing’s private cause a lot of them.*

[Helena, 6-month-old female, uses one IFA, ceased another at 6 wk]

One participant stated:

*I assume that it’s all aggregated data for sale*.

[Helena, 6-month-old daughter, uses one IFA, ceased another at 6 wk]

For some mothers, the nature of the data input reduced their concerns in relation to privacy:

*I don’t feel as though I’m providing data that’s too personalised, that’s a threat I suppose to have on there. It’s just times*.

[Bernadette, 5-month-old daughter uses two IFAs]

Lucia shared a similar view, expressing little concern over data related to feeding or diaper changes:

*I don’t really think about the data in relation to the baby stuff. [...] How many times I’m getting my boob out or changing nappies, I don’t really care if anyone knows.*

[Lucia, 5-month-old son, uses two IFAs]

Many mothers described feeling resigned to the ubiquity of data collection across digital platforms, recognizing that similar practices occur on widely used services like Facebook and Google.

*[...] we’re so used to it (refers to data used for marketing) [...] once you search for one thing they feed you 10 other things related to it and I still use Facebook, I still use Google and everything so I probably would still use it but I would feel a little bit strange or uncomfortable with it, I guess*.

[Cassandra, 6-month-old daughter, uses one IFA]

Hyacinth echoed these views, emphasizing that data collection through IFAs does not worry her because data are being collected from phones in general. She explained:

*[...] I don’t really care that much either. I think with data collection our data’s being collected anywhere. Any time we pick up our phone, our data’s being collected from somewhere, and I guess that kind of data that I’m entering into the app [...] I don’t really see how they can use that in any – they can use it however they want; it’s not really affecting anyone*.

[Hyacinth, 5-month-old daughter, uses two IFAs, ceased another]

Interestingly, the wider policy environment shaped how some participants thought about privacy issues and helped normalize data sharing. This was highlighted in Babette’s comment, which contrasted data privacy laws between the EU and Australia.

*I really hate the EU data privacy laws...you have to repeat your information all the time because of data protection...that’s one of the reasons why I decided to move back to Australia*.

[Babette, 5-month-old female, uses one IFA, ceased another]

As several participants explained, this behavior reflected not ignorance but a pragmatic response to the demands of parenting and perceived inevitability of data sharing in digital environments. Mothers normalized data sharing, and strict rules were sometimes seen as frustrating rather than protective, reinforcing that surveillance of data was perceived as a normal part of everyday digital life.

#### Subtheme 2: Benefits Outweigh Risks

Participants spoke about their willingness to forgo privacy concerns in exchange for higher levels of perceived convenience. Some participants differentiated between digital apps, tolerating IFAs while expressing greater concerns about other platforms like Facebook and Instagram.

The majority of mothers described focusing more on the app’s utility than on the potential data privacy violations. The utility of IFAs in health care contexts and everyday parenting often outweighed privacy concerns. Patty highlighted their value, stating:

*When I would go to the child health nurse checkup, I would have all the data and there ready to go. I knew what my trends were, what the averages were*.

[Patty, 8-month-old female, uses one IFA, ceased another]

The same participant reframed targeted advertising as supportive:

*Being suggested a product, I think that’s helpful for mum*.

[Patty, 8-month-old female, uses one IFA, ceased another]

Cassandra expressed that despite knowing that her data were being collected, she would continue to use an app as well:

*I’ve been reading a lot about the privacy of data in those apps which I also used when I was trying and pregnant...Obviously it’s not such a concern that it’s stopping me using it ‘cause I’m still using it and putting in heaps and heaps and heaps of data*.

[Cassandra, 6-month-old female, uses one IFA (INT1)]

Bernadette’s comments provide further insight. In the early stages of using an IFA, she wasn’t focused on privacy concerns as her primary goal was to reduce the mental load of caregiving:

*In the beginning it probably wasn’t something that I was thinking of; I was looking for something to take a mental load off*.

[Bernadette, 5-month-old daughter uses two IFAs]

However, after experiencing a data breach that compromised her online accounts, she now views data privacy differently, though it wasn’t a priority when she first started using the app.

Another mother added how common it is to overlook privacy considerations, reflecting on the convenience that IFAs provide and on prioritizing their baby’s needs:

*Yeah. I don’t know if it would have stopped me from using it or not, but I don’t think it would have stopped me from using it.*

[Paris, 6-month-old son one IFA ceased at 5 months]

Elspeth and Felicia have never used an IFA but shared a similar experience, explaining that during the intense period of feeding, privacy was not a major concern and was not a consideration regarding whether to use an IFA or not. Although they did not use an IFA, they relied on other online resources. She said:

*I haven’t really thought about it collecting information so that probably hasn’t been a primary concern for me*.

[Elspeth, 1-month-old son, never used IFAs]

*I wasn’t thinking about privacy and data collection [...] I was thinking about what my baby needed from me at that time*.

[Elspeth, 1-month-old male, never used IFAs (FG)]

*I don’t think I even thought about any concern about data privacy as something; again, I just think about what my baby needs.*

[Felicia, 7-month-old female twins, has never used IFAs (FG)]

In retrospect, Elspeth was not sure her awareness of privacy concerns would have changed her behavior, noting that she uses other subscription services without much concern for data collection.

*...It’s such a complex world that’s changing so much that I don’t fully understand it enough. So, I don’t know how worried I am about it at this point, I suppose.*

[Elspeth, 1-month-old male, never used IFAs (FG)]

Others, like Semra, who used 2 IFAs, rationalize that if their data are used for improving services or targeted marketing, they would still use the app if the benefits outweigh the risks.

*I think they use it to sell me something [...] that’s fine if they found something that was helpful for me.*

[Semra, 8-month-old daughter, uses two IFAs]

Overall, while some awareness of privacy risks related to IFAs exists, most mothers do not engage deeply with the terms and conditions. Concerns about data privacy, while present, are often outweighed by the perceived utility of the IFAs, leading mothers to continue using them despite reservations. As Bernadette illustrates, the focus remains on immediate caregiving needs, and the complexity of digital privacy in today’s world makes it difficult for parents to fully assess or act on these concerns and rarely deter IFA use.

Another mother added that data sharing that led to greater good through research may justify data collection or their personal information being tracked:

*...] if they took all that data and used it for research purposes then I’d be fine with it because it’s not like it’s identified data, it’s all deidentified, really, they don’t have anything*.

[Semra, 8-month-old daughter, uses two IFAs]

This suggests that while some participants also demonstrated varying levels of concern over privacy issues among different digital apps, IFAs were perceived as being less invasive compared with other apps.

Collectively, both themes illustrate a tension between risk awareness and acceptance, underscoring how perceived utility often outweighs concerns about surveillance and data trails. While it is true that many users of smart and digital apps commonly skip reading terms and conditions, our findings extend understanding where mothers’ quotations revealed how parents specifically interpret and negotiate such practices in the context of infant-feeding apps, as shown in the next section. For example, participants discussed privacy concerns and risk management in relation to their children’s health, which reframes a general behavior into a context-specific act. Thus, while the behavior itself may be widespread, its meaning and implications in the domain of infant care are unique to this group.

### Theme 2: Risk Mitigation Behaviors

Under this theme, mothers reflected on the measures they employed to mitigate their concerns around data privacy. This included various measures, such as selective disclosure when participants withheld or altered personal details, or avoiding child images or using shortened names.

Some mothers adopted privacy-conscious behaviors such as using pseudonyms or incomplete personal information (eg, incorrect birth dates) to reduce their exposure.

*[...], I tend to not put anything majorly personal on these apps so I don’t have my real name – I have my name but my shortened name so just Patty and [Joey] like I don’t put very big identification things on there for that reason probably*.

[Patty, 8-month-old daughter, uses one IFA, ceased another at 4 mo]

Similarly, Lucia emphasized her ability to control disclosure, stating:

*We’re pretty careful with any forms we fill out for the hospital... say don’t add to my health record and things ‘cause it just feels a bit creepy’*.

[Lucia, 5-month-old male, uses two IFAs]

Several other mothers, such as Helena, Lauretta, and Semra, shared actions that reflected privacy-conscious behavior and increasing awareness of data vulnerabilities and concern over potential surveillance of their data:

*[....], they won’t let you use it unless you put in the name and the date of birth and that kind of thing, I just make it up, I use something different cause I’m like that doesn’t matter. If they’re going to give age-based recommendations then I’ll change it by two days or something like they just don’t need that info*.

[Helena, 6-month-old daughter, uses one IFA, ceased another at 6 wk]

*[....], I’m also probably mindful when I am putting things online that I don’t put anything that’s too disclosing about where I live or anything like that so they’re not going to be getting any data that I wouldn’t care if anyone knew*.

[Lauretta, 6-month-old son, uses two IFAs]

Another participant echoed:

*I probably didn’t really care too much for Huckleberry and Solid Starts, things that I don’t even put too much of my personal information in so it’s not linked to my Facebook or any of my applications and I can choose whether I put in her real name, her fake name, a photo*.

[Semra, 8-month-old daughter, uses two IFAs]

Overall, mothers’ awareness of privacy risks generally did not strongly influence their behavior, and many admit they haven’t given much thought to the potential risks. As Morgana noted, her concerns about data privacy increased after a Facebook hack, leading her to reduce her online activity:

*I’ve pulled back on sharing a lot of information [...] I’ve deleted a couple of apps and signed out of them because of it*.

[Morgana, a 7-month-old male, uses one IFA (INT1)]

Our findings suggest a dynamic interplay between awareness of surveillance, leading to selective disclosure and deidentification. It shows that mothers acknowledged vulnerabilities and privacy concerns, yet they actively negotiated their engagement and adopted everyday privacy practices that allowed continued use with unease.

## Discussion

### Principal Findings

In this study, we explored mothers’ understanding of the terms and conditions associated with IFAs and their data privacy concerns. The first emerging theme was awareness and acceptance of terms and conditions. Our findings show that participants demonstrated awareness that engaging with IFAs involved data collection and potential privacy risk. Despite this, many described accepting terms and conditions without reading these in detail, often citing limited time or the perceived inevitability of such practices. For some, this acceptance was framed as a trust in implied digital security, while others expressed resignation, noting that data collection is now an unavoidable aspect of online activity. A few participants articulated the belief that “everyone‘s data are up for sale,” reflecting a broader recognition that privacy compromises are inherent in the use of digital devices and apps.

Building on these insights, it is important to note that control over data remains a fundamental right. The Australian Competition and Consumer Commission’s report [20] emphasized reforms to ensure consumers are informed and protected in how their data are used (p. 3). Privacy is negotiated through balanced disclosure with protection [21], but information shared through IFAs may persist, be widely distributed, and be repurposed in unintended ways [22-25]. Our findings show that while some participants acknowledged the potential of their data to be distributed or reused, most participants expressed acceptance of such practices as an unavoidable aspect of digital engagement. In Australia, the Privacy Act sets out 13 Australian Privacy Principles based on a notice and consent model, which places responsibility on individuals to manage their own personal information and to interpret complex privacy policies [26]. For many participants, reviewing such policies was unrealistic due to time constraints or trust in implied protections, a finding consistent with prior research [27]. These findings underscore the limits of privacy self-management and reinforce calls for a mandated “privacy by design” approach, embedding protections into platform development from the outset [28].

Digital privacy is critical for all users of platforms that collect personal data and is widely regarded as essential for protecting consumers from unwanted access and misuse [29].

Privacy carries a positive value, while invasion of privacy is seen as a violation of a protected interest. In this way, participants in this study reflected the broader social narrative that privacy should be protected in digital space [30]. This is consistent with the right to privacy in the digital age that was affirmed by the United Nations General Assembly and the Human Rights Council [31], requiring that any interference meet the principles of legality and proportionality, while noting that emerging technologies, such as artificial intelligence, can affect this right [32].

Despite these protections, many remain resigned to threats from big data, cloud computing, and profiling [33]. Participants in the study accepted that app-collected data could be commodified, expecting it might be used or sold. This raises complex questions about when online data are classified as public or private, whether their reuse constitutes intervention without consent, and how individuals’ privacy can be safeguarded [34].

The second emerging theme was risk-mitigation behaviors, where participants described exercising a degree of control over the data they shared by adopting strategies to mitigate perceived risks. These included using pseudonyms rather than real names and obfuscating identifiers such as date of birth. Participants also constructed personal boundaries around what information they considered acceptable to disclose. For example, not sharing visual images was important to one mother, while another noted that the type of information shared, such as the number of feeds or nappy changes, did not feel too personal to share more widely.

Overall, participants perceived the benefits of engaging with IFAs as outweighing the potential risks. Many described a willingness to forgo privacy concerns in exchange for higher levels of perceived convenience. The majority of mothers described focusing more on the mobile app’s utility than on the potential data privacy violations. Trust in the security of digital systems and a belief in having some control over the information shared contributed to this sense of acceptance. These findings highlight the complex interplay between utility and privacy trade-offs and are discussed in relation to current debates on data privacy in online environments.

Some participants in this study believed they could protect their privacy by altering key identifiers, such as date of birth. This highlights which types of information about themselves were considered more sensitive than behavioral data like feeding or changing routines. Obscuring personal information is not new and is practiced offline as well [35], with individuals deciding how much to reveal in different contexts.

Online, people sometimes create “Amateur Artists” [36] to protect their privacy. These disguises can range from complete reinvention [37] to small changes, like those seen in this study, where mothers adjusted details but still felt their data reflected real experiences while giving them some protection. Not everyone supports this practice. Zuckerberg argues that having more than one identity shows a lack of integrity and that people should present a single, authentic self online [38,39]. Similarly, Google’s Eric Schmidt claimed that “Innocent people have nothing to hide” [40], suggesting privacy concerns are unnecessary.

Our finding is that self-disclosure is complex, with different views on what counts as personal and sensitive information. Literature highlights dimensions such as the depth and value of what is shared [41]; for example, demographic details may be seen as less sensitive than private photos or emotions. However, individuals may share private data unknowingly due to limited risk awareness [42,43], lack of knowledge about ways to protect data [44], or not being aware of how data are distributed postsubmission [45].

Consent remains central to the use of digital data; reliable collection depends on mutual respect and trust between parties [30]. IFAs that require login, membership, or subscription create a sense of “perceived privacy,” yet most participants in this study did not expect their data to be shared beyond the app and may not have realized this was possible [46]. Consistent with earlier reports, concerns relating to the security of their data have led some users to stop or delete IFAs [47,48], but research on the link between privacy and security in mobile apps remains limited [49,50]. Evidence shows that privacy risks reduce trust in app security, while clear policies can strengthen it. In the health context, privacy can only be assured where individuals control their information and prevent unnecessary disclosure [51,52]. Digital safety is increasingly recognized as essential, particularly for children and young people [53]. Building digital safety requires self-efficacy, knowledge, and skills related to managing risks. Awareness of digital safety can assist parents in adopting strategies to safeguard themselves and their families [53].

The findings of this study were limited by the restricted demographic data collected and the sample profile. This study was limited to English-speaking Australians, breastfeeding mothers recruited mainly via Facebook, which may limit diversity and generalizability. This recruitment strategy may have introduced selection bias and limited the diversity of perspectives, particularly among culturally and linguistically diverse parents or those with lower levels of digital literacy. Consequently, the findings should be interpreted within the context of the participant profile and may not fully reflect the experiences of caregivers from different backgrounds.

Another limitation is the absence of information on socioeconomic and educational data, which further constrains contextual interpretation. These factors may have influenced perspectives on digital privacy and engagement with IFAs, and their absence restricts the ability to fully contextualize findings. However, the study was designed to focus on parents’ experiences and perceptions of IFA use rather than to compare outcomes across demographic groups. While this approach provided valuable insights into usage patterns and parental perspectives, future studies would benefit from including broader demographic variables to capture how diverse backgrounds may shape digital practices and privacy concerns. Use of mixed methods and longitudinal approaches can enrich data by combining both qualitative and quantitative insights into privacy literacy and behaviors.

### Conclusions

Parents of young children carry dual responsibilities as advocates and primary caregivers [54], and many now rely on digital apps, such as IFAs, to track and manage health behaviors. Breastfeeding as a socially and emotionally charged practice has become increasingly mediated through digital tools, reflecting the demand for accessible, time-sensitive information [55,56]. While such technologies present opportunities for increased access to education and health promotion [57], they also introduce risks that require careful negotiation [53]. Our study shows that mothers’ engagement with IFAs is shaped by 2 key dynamics: awareness and acceptance of terms and conditions and risk-mitigation behaviors. This paper has highlighted the varying levels of awareness among mothers of how data about themselves and their child can be used and their expectations of how personal information will be kept confidential and secure through their engagement with a breastfeeding mobile app. The findings highlight the need for action toward privacy by design, transparency in privacy policies, and nonalienation in relation to the use of data. The use of IFAs reveals a paradox: mothers acknowledge the inevitability of data collection, yet actively resist through boundary-setting and risk-mitigation. Embedding privacy by design and fostering genuine trust in IFAs is essential to protect both parental and child well-being

Our findings highlight the importance of transparent privacy policies and respect for contextual integrity and user-centered safeguards that enhance digital safety, protect against hidden risks, and ensure accountability in data use.

## Abbreviations

FG: focus group
IFA: infant feeding app
